# Effects of adding R-BoT + plus verticalization-based robotic locomotor training to conventional rehabilitation in patients with subacute stroke: a randomized controlled trial

**DOI:** 10.1186/s12984-026-02053-z

**Published:** 2026-06-23

**Authors:** Kyung Han Kim, Yoo Kyeong Han, Jung Eun Son, Arum Jeon, Hyo Been Lee, Miae Lee, Seong Kyu Noh, Hak Byung Kim, Eo Jin Park, Seung Ah Lee, Sung Joon Chung, Dong Hwan Kim, Seung Don Yoo

**Affiliations:** 1 Department of Rehabilitation Medicine, Kyung Hee University Hospital at Gangdong, Seoul, 05278 Korea; 2 Healthcare Big-Data Center, Medical Science Research Institute, Kyung Hee University Hospital at Gangdong, Seoul, 05278 Korea; 3 Cotras Industry, Daegu, 41500 Korea

**Keywords:** Stroke, Subacute, Gait, Robotic rehabilitation, R-BoT + plus, Functional ambulation category, Trunk control

## Abstract

**Background:**

Early recovery of gait and trunk control during the subacute phase after stroke is crucial for long-term functional outcomes. Robotic-assisted rehabilitation enabling upright positioning, weight-bearing, and repetitive stepping-like movements has emerged as a promising adjunct to conventional therapy. The primary aim of this study was to evaluate whether adding R-BoT + plus verticalization-based robotic locomotor training to conventional inpatient rehabilitation leads to greater improvement in gait independence than conventional rehabilitation alone. The secondary aims were to assess the effects of the intervention on trunk control, cognitive function, activities of daily living (ADL), balance, and lower-extremity motor function.

**Methods:**

This single-center, prospective, randomized controlled trial was conducted in an inpatient rehabilitation setting and enrolled patients with subacute stroke and severe gait impairment. Participants were randomly assigned to receive either R-BoT + plus verticalization robotic locomotor training combined with conventional rehabilitation or conventional rehabilitation alone. Both groups received conventional rehabilitation five times per week for 4 weeks. The experimental group also received R-BoT + plus verticalization-based robotic locomotor training with the same frequency and duration (20 sessions; 30 min per session). Outcomes were assessed at baseline (preintervention) and postintervention, with a 3-month follow-up for gait independence.

**Results:**

Patients who received R-BoT + plus training demonstrated significantly greater improvement in the Functional Ambulation Category than the control group. Mixed-effects modeling showed sustained benefits favoring the experimental group at postintervention and at 3-month follow-up. Although the overall change in Trunk Control Test scores did not differ significantly between groups, an exploratory baseline-adjusted robust regression suggested that patients with lower baseline trunk control experienced significantly greater post-intervention improvement in the experimental group. Other functional outcomes, including cognition, balance, ADL, and lower-extremity motor function, improved in both groups without significant between-group differences.

**Conclusions:**

R-BoT + plus verticalization-based robotic locomotor training provides additional benefits for gait independence and trunk control when added to conventional rehabilitation in patients with subacute stroke, particularly among those with greater initial impairment.

*Trial registration*: This study was prospectively registered at the Clinical Research Information Service (CRiS), Republic of Korea (registration number: KCT0010040).

**Supplementary Information:**

The online version contains supplementary material available at https://doi.org/10.1186/s12984-026-02053-z.

## Background

Stroke remains one of the leading causes of long-term disability worldwide, and impairments in gait and balance are among the most disabling sequelae affecting independence, participation, and quality of life. A substantial proportion of stroke survivors experience persistent limitations in ambulation, with approximately three-quarters reporting gait dysfunction during recovery [1]. Accordingly, the restoration of safe, independent walking is a central goal of stroke rehabilitation.

The subacute phase after stroke, generally defined as the period from several days to months after onset, is characterized by heightened neuroplasticity and increased responsiveness to rehabilitation [2, 3]. During this time window, intensive, task-specific, and repetitive training has been shown to exert a disproportionate influence on long-term functional outcomes compared with later stages of recovery [4]. Early gait rehabilitation incorporating upright positioning, weight-bearing, and stepping-like movements plays a crucial role in promoting locomotor recovery and preventing secondary complications. In clinical practice, these principles are traditionally implemented through various therapeutic activities, including level-ground walking, stair-climbing, and outdoor ambulation to enhance functional mobility.

However, many patients in the early subacute phase—particularly those with severe motor impairment, insufficient trunk control, or Functional Ambulation Category (FAC) scores of 2 or lower—are unable to participate safely or effectively in these conventional overground gait training activities. Therapist-assisted walking in these patients is often limited by safety concerns, high physical demands on therapists, and difficulty achieving sufficient training intensity and repetition. These challenges have driven the development of robotic and electromechanical rehabilitation devices to facilitate early mobilization and gait-related training under controlled conditions.

Robot-assisted locomotor interventions have increasingly been adopted as an adjunct to conventional stroke rehabilitation, enabling high-intensity, repetitive, and task-oriented lower-limb movements while reducing the physical burden on therapists. In particular, conventional robot-assisted gait training (RAGT) systems are typically designed to facilitate full gait cycles under upright conditions, often using treadmill-based or exoskeleton devices. Previous randomized controlled trials and systematic reviews have reported beneficial effects of such approaches on walking ability and functional outcomes after stroke; however, results have varied depending on stroke chronicity, baseline severity, and device-specific characteristics [5, 6].

For patients who cannot tolerate full upright posture or independent walking early after stroke, alternative strategies that combine gradual verticalization, partial weight-bearing, and cyclic lower-limb movements may provide a practical means of initiating locomotor-related training. These approaches allow early exposure to upright loading and stepping-like activity while maintaining physiological stability, and may be particularly relevant for patients with severe gait impairment during the subacute phase. Such interventions are more appropriately conceptualized as robot-assisted stepping or verticalization-based pre-gait training, rather than conventional RAGT [7]. Nevertheless, evidence remains limited regarding the clinical effectiveness of specific device-based interventions in this population.

**Fig. 1:**
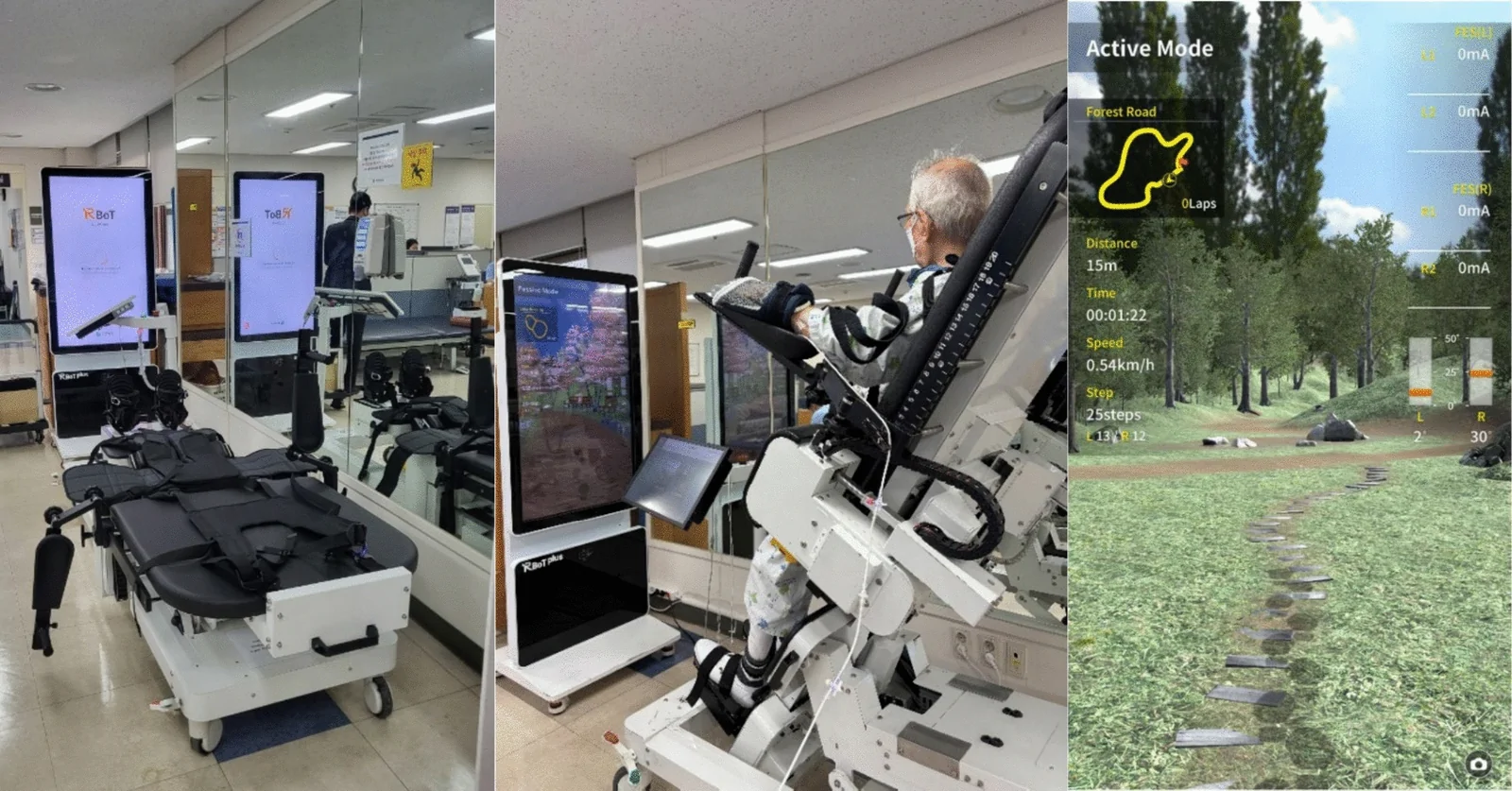
R-BoT + plus system

R-BoT + plus system (CoTras, Daegu, Republic of Korea) is a tilt-table-type gait-assisted robotic rehabilitation device designed to support early mobilization in patients with limited standing or walking ability. It enables gradual verticalization from supine to upright, adjustable body-weight support, and repetitive stepping-like movements of the lower limbs (Fig. 1). The system enables gradual verticalization from a supine position and delivers repetitive lower-limb movements across multiple training modes, tailored to each patient’s capacity. Visual feedback through task-oriented virtual environments, along with optional integration with functional electrical stimulation (FES), is intended to enhance patient engagement, sensorimotor integration, and motor learning during training.

Given the importance of early gait recovery and trunk control for long-term functional independence, it is essential to determine whether adding R-BoT + plus training to conventional rehabilitation provides additional clinical benefit in patients with subacute stroke and severe gait impairment. Therefore, the primary aim of this randomized controlled trial was to evaluate whether adding R-BoT + plus verticalization-based robotic locomotor training to conventional inpatient rehabilitation leads to greater improvement in gait independence, as measured by the FAC, compared with conventional rehabilitation alone. The secondary aims were to assess the effects of the intervention on trunk control, cognitive function, activities of daily living (ADL), balance, and lower-extremity motor function.

## Materials and methods

### Study design

**Fig. 2:**
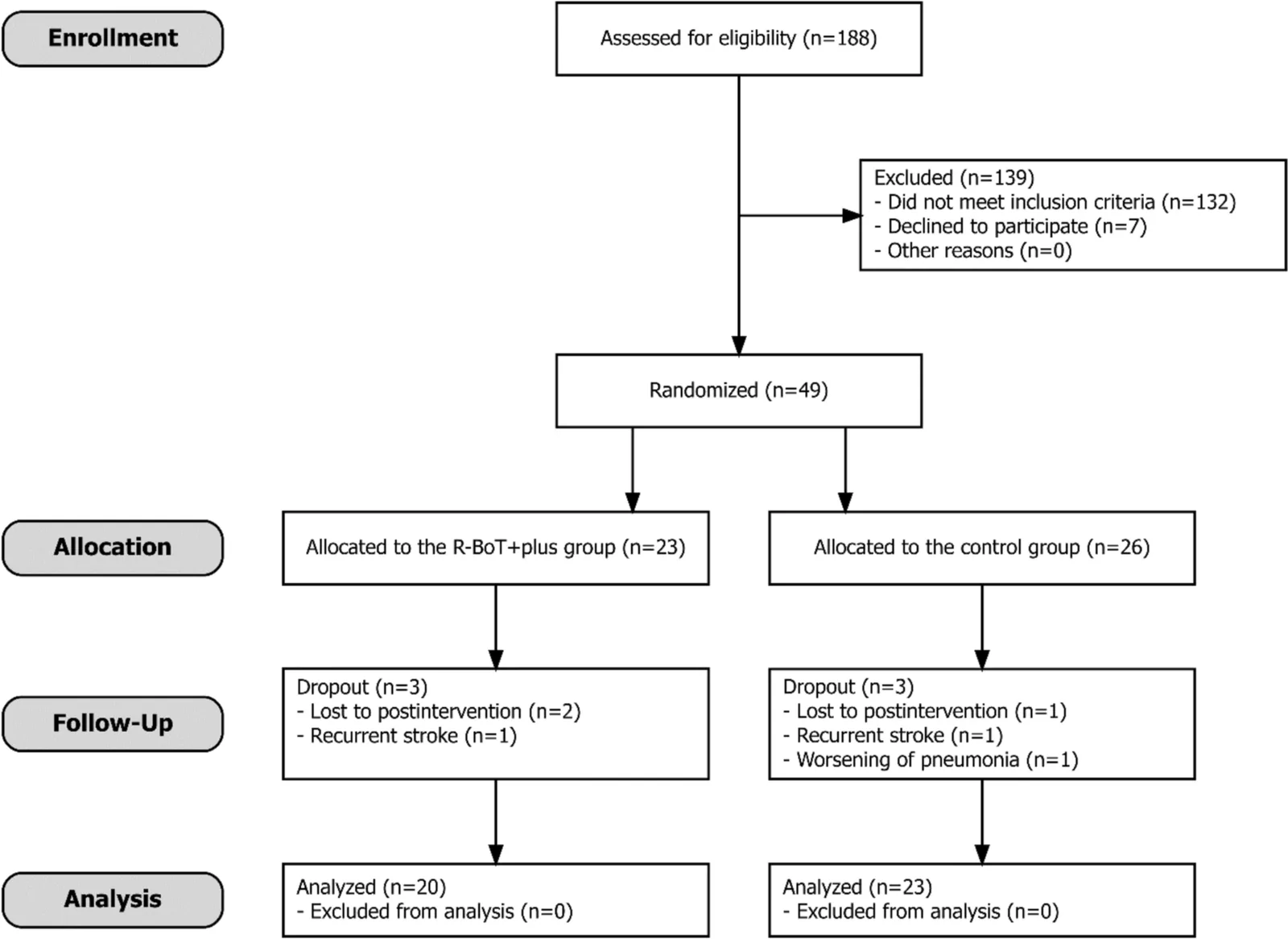
CONSORT flow diagram of the study

This study was a single-center, prospective, randomized, assessor-blinded, parallel-group controlled trial conducted in patients with subacute stroke admitted for inpatient rehabilitation. Between September 13, 2024, and December 31, 2025, consecutive patients with stroke admitted to the Department of Rehabilitation Medicine at Kyung Hee University Hospital at Gangdong were assessed for eligibility (*n* = 188). Patients who met the predefined inclusion criteria were randomly allocated to either an experimental group receiving R-BoT + plus verticalization-based robotic locomotor training in addition to conventional rehabilitation or a control group receiving conventional rehabilitation alone (Fig. 2). Assessments were performed at baseline (pre-intervention), immediately after the intervention period (post-intervention), and at 3-month follow-up for FAC.

### Participants

The inclusion criteria were the following: age ≥ 19 years; diagnosis of ischemic or hemorrhagic stroke confirmed by computed tomography or magnetic resonance imaging; subacute phase, defined as approximately 7 days to 6 months after stroke onset at the time of enrollment; unilateral hemiparesis or hemiplegia involving the lower extremity; baseline FAC ≤ 2, indicating inability to walk independently or requirement for substantial assistance in ambulation; patients with independent ambulation prior to stroke onset; medically stable vital signs and cardiovascular status, such that upright positioning and R-BoT + plus training were considered safe by the treating physician; ability to follow simple one- or two-step verbal commands and to communicate basic needs; provision of written informed consent by the patient or a legally authorized representative.

Participants with any of the following conditions were excluded: unstable or severe cardiovascular disease (e.g., unstable angina, myocardial infarction within the past 3 months, decompensated heart failure, clinically significant arrhythmia) that would make upright or gait training unsafe; severe respiratory disease (e.g., chronic respiratory failure requiring continuous oxygen therapy, resting dyspnea) limiting exercise tolerance; recent lower-limb fracture, major joint surgery, or severe joint deformity or contracture precluding safe weight-bearing or stepping movements; marked lower-limb spasticity (e.g., Modified Ashworth Scale ≥ 3) that would interfere with the safe application of the device; other major central nervous system conditions (e.g., Parkinson disease, multiple sclerosis, spinal cord injury) that could confound outcomes; severe cognitive impairment, persistent reduced consciousness, or delirium precluding cooperation with training and assessments; severe visual disturbance, neglect, or other perceptual deficits that would severely compromise safety during training (investigator’s judgment); severe psychiatric or behavioral problems, lack of cooperation, pregnancy, or any other condition deemed by the investigator to pose unacceptable risk or make participation inappropriate.

### Randomization and blinding

After baseline assessment, participants were randomized to the experimental or control group (1:1 ratio) using a computer-generated random sequence. Allocation was concealed using sealed, opaque envelopes opened by an independent coordinator. Because of the nature of the intervention, participants and treating therapists could not be blinded to group allocation. However, outcome assessors were not involved in treatment delivery and were instructed to remain unaware of group assignment wherever feasible.

### Interventions

All participants received conventional inpatient stroke rehabilitation, including physiotherapy and occupational therapy, delivered according to institutional protocols. Conventional therapy typically focuses on range-of-motion exercises, muscle strengthening, task-specific gait and balance training as tolerated, and ADL training.

#### Control group: conventional rehabilitation alone

Participants in the control group received conventional inpatient rehabilitation only, consisting of physiotherapy and occupational therapy delivered five times per week for 4 weeks. Because most participants had severe gait impairment at baseline (FAC 0–2), conventional rehabilitation emphasized preparatory locomotor rehabilitation rather than independent overground ambulation alone. Each physiotherapy session included approximately 30 min of gait- and lower limb-focused therapy, comprising supported standing, trunk control exercises, weight-shifting practice, therapist-assisted stepping activities, balance training, lower-extremity strengthening, task-oriented functional training, and overground gait training as tolerated. Physiotherapy also included verticalization and standing exercises using a tilting table, standing frame, and stall bar standing according to each participant’s functional status and tolerance. In addition, FES was applied to patients with lower-extremity muscle strength of ≤grade 3 on the Manual Muscle Test (MMT). The specific content and progression of therapy were individualized according to each participant’s functional status, tolerance, and clinical safety. In contrast, participants in the experimental group received the same conventional rehabilitation program in addition to R-BoT + plus training, rather than replacing conventional gait training.

#### Experimental group: R-BoT + plus plus conventional rehabilitation

The participants allocated to the experimental group received robot-assisted gait training using the R-BoT Plus system, in addition to the same conventional inpatient rehabilitation program provided to the control group. Conventional rehabilitation consisted of physiotherapy and occupational therapy delivered five times per week for 4 weeks, including approximately 30 min of gait- and lower limb-focused therapy per session.

Robot-assisted training was performed using the R-BoT Plus system and followed a predefined intervention parameter including:

- progressive increase of tilt angle and weight-bearing as tolerated;
- bilateral stepping movements with speed and range adapted to patient tolerance and safety;
- close monitoring of blood pressure, heart rate, and symptoms during training.

Specifically, stepping speed was initiated at 15–20 steps/min and progressively increased toward a target of 40 steps/min according to patient tolerance. Hip joint excursion was initiated at 30° and gradually increased to a target angle of 45°. The tilt angle was initially set at 30° and progressively increased by 10° per session in the absence of orthostatic hypotension, with a target tilt angle of 80.5°.

R-bot + plus sessions were administered 5 sessions per week for four 4 consecutive weeks, with each session lasting 30 min, for resulting in a total of 20 sessions. Session frequency, duration, and core training parameters were delivered using predefined and standardized intervention parameters and applied consistently across participants in the experimental group.

FES was routinely applied to the paretic side of all participants who exhibited a muscle strength of ≤grade 3 on the MMT. The electrodes were specifically positioned over the tibialis anterior to facilitate ankle dorsiflexion and the quadriceps femoris to support knee extension during stepping movements. The FES settings were standardized across all participants in the experimental group, with an amplitude level of 15 (maximum level: 15), pulse width level of 8 (maximum level: 8), and frequency level of 9 (maximum level: 9).

In addition, real-time visual feedback was provided through a monitor-based virtual environment integrated into the R-BoT + plus system (Fig. 1). Participants selected either the “Lake Road” or “Forest Road” mode, in which a first-person walking simulation was displayed during training. The interface continuously provided visual feedback regarding training performance, including step count, walking speed, and bilateral hip joint angles. The visual feedback component was intended to promote active participation, task-oriented motor practice, and sensorimotor integration during repetitive stepping training. No additional verbal cueing related to gait performance was provided by the therapist during the intervention.

### Outcome measures and assessment schedule

All assessments were performed by trained therapists who were not involved in delivering the R-BoT + plus training. Assessments were conducted at baseline (preintervention; all outcome measures), at postintervention (immediately after the completion of the inpatient rehabilitation/intervention period; all outcome measures), and at 3-month follow-up (FAC only).

The primary outcome is Functional Ambulation Category (FAC), a 6-point ordinal scale (0–5) that assesses the level of physical support required for ambulation, with higher scores indicating greater gait independence. FAC is widely used in stroke populations and has demonstrated good inter-rater reliability and clinical validity in assessing walking ability [8].

The secondary outcomes are the following: Trunk Control Test (TCT), which evaluates trunk control in rolling, sitting, and balance tasks, with scores ranging from 0 to 100 and higher scores indicating better trunk control [9]; Korean Mini-Mental State Examination (K-MMSE), which evaluates global cognitive function [10]; Korean Modified Barthel Index (K-MBI), a 10-item measure of independence in basic ADL, with higher scores indicating greater independence [11]; Berg Balance Scale (BBS), a 14-item performance-based measure of static and dynamic balance, with higher scores indicating better balance [12]; lower-extremity Fugl-Meyer Assessment (FMA), which evaluates motor function using the combined lower-extremity motor and coordination/speed subscores, with higher scores indicating better motor performance [13]. All outcome measures have been widely used in patients with stroke and have demonstrated acceptable reliability and validity for assessing functional recovery during the subacute phase [14].

### Statistical analyses

The sample size was calculated using G*Power version 3.1.9.7 (Heinrich-Heine-Universität Düsseldorf, Germany). [15] The expected effect size was estimated based on a previous randomized controlled trial of robot-assisted gait training in patients with stroke by Mustafaoglu et al. [16] With a two-sided α level of 0.05 and a statistical power of 0.95, a minimum of 20 participants per group was required. Accounting for an anticipated 30% dropout rate, the target enrollment was set at 52 participants. The calculation was based on the planned primary outcome analysis; thus, analyses of secondary outcomes were considered exploratory. Recruitment concluded with 49 randomized participants when the predefined funding-supported study period ended. Although the planned target enrollment was not fully achieved, the final sample exceeded the minimum number of participants required for the primary outcome analysis.

Baseline characteristics and outcome measures were summarized as mean ± standard deviation (SD) for continuous variables and as frequencies with percentages for categorical variables. Continuous scales were presented as mean ± SD to facilitate clinical interpretability and comparison with previous literature. Between-group comparisons of baseline characteristics were performed using the chi-square test or Fisher’s exact test for categorical variables, and the independent-samples *t* test for continuous variables; when normality was not satisfied, the Wilcoxon rank-sum test was used.

The primary outcome, the longitudinal change in FAC scores, was analyzed using a linear mixed-effects model [17]. The model included treatment group as the between-subjects factor, time as the within-subjects factor, and the group-by-time interaction as the primary term of interest, with a participant-specific random intercept to account for within-subject correlation. Model-estimated FAC means and standard errors (SEs) were derived, and post hoc model-based contrasts were used to compare between-group differences in change from baseline at postintervention and 3-month follow-up. The *p* values for these post hoc FAC contrasts were adjusted for multiple comparisons using the Holm method [18].

Individual-level transitions in FAC categories from baseline to postintervention and 3-month follow-up were visualized using Sankey diagrams stratified by group. To provide a statistical comparison corresponding to these diagrams, individual-level FAC changes from baseline were categorized as ≥+2, +1, 0, and ≤−1, and the distributions of FAC change categories were compared between groups using Fisher’s exact test because of sparse cell counts.

For outcomes assessed at baseline and postintervention, individual-level change scores were calculated as postintervention minus baseline values. These change-score analyses were considered supportive for FAC and exploratory for secondary outcomes. Between-group comparisons of change scores were performed using the Wilcoxon rank-sum test due to potential non-normality. To address multiplicity across secondary outcomes, false discovery rate (FDR) *q*-values were calculated using the Benjamini–Hochberg method [19].

To investigate whether baseline trunk control moderated the treatment effect on postintervention TCT scores, an exploratory robust linear regression model was used. In this model, postintervention TCT score was the dependent variable, and treatment group, baseline TCT score, and the group × baseline TCT interaction were included as independent variables. Baseline FAC was not included in this model. Robust regression was performed using M-estimation with Huber weighting to mitigate the influence of influential observations and outliers identified during model diagnostics [20]. To facilitate clinical interpretation, conditional treatment effects (R-BoT + plus − control) were derived from the model at prespecified baseline TCT values of 25, 50, and 75, representing clinically relevant severity levels.

Missing outcome data were not imputed. Pre–post change-score analyses included only participants with complete data at both time points. For the primary FAC linear mixed-effects model, all available longitudinal measurements were utilized without imputation. Analysis-specific sample sizes are provided in the relevant tables and figure legends, where applicable.

All analyses were performed using R version 4.2.3 (R Foundation for Statistical Computing, Vienna, Austria). All statistical tests were two-sided, and *p* < 0.05 was considered statistically significant.

## Results

### Participant characteristics

A total of 49 participants were randomized in this study: 23 to the experimental group, receiving R-BoT + plus combined with conventional rehabilitation, and 26 to the control group, receiving conventional rehabilitation alone. An overview of participant flow, including any dropouts and reasons, is presented in a CONSORT flow diagram (Fig. 2).

In the experimental group, three participants discontinued the study: two were lost to post-intervention assessment, and one withdrew due to recurrent stroke. In the control group, three participants discontinued the study, including one lost to post-intervention assessment, one due to recurrent stroke, and one due to worsening of pneumonia.

Ultimately, 20 participants in the experimental group and 23 participants in the control group completed the post-intervention assessment and were included in the final analysis. No serious adverse events directly attributable to the R-BoT + plus device were observed during the intervention period.

Baseline characteristics are summarized in Table 1. Baseline FAC, K-MMSE, TCT, K-MBI, and BBS scores were similar between groups, and most participants had FAC scores of 0–1, indicating an inability to walk independently at baseline.

**Table 1:** Baseline characteristics of the participants

Characteristics	R-BoT + plus (*n* = 20)	Control (*n* = 23)	*p* value
Demographics			
Sex			0.246
Male	14 (70.00)	11 (47.83)	
Female	6 (30.00)	12 (52.17)	
Age (years)	62.95 ± 13.24	65.26 ± 15.69	0.608
Height (cm)	168.35 ± 8.13	166.01 ± 8.35	0.360
Weight (kg)	74.35 ± 12.91	68.33 ± 13.88	0.083
BMI (kg/m^2^)	26.34 ± 3.54	24.71 ± 4.24	0.053
Time since stroke (days)	21.15 ± 8.63	29.09 ± 19.64	0.168
Lesion type			>0.999
Hemorrhagic	7 (35.00)	7 (30.43)	
Ischemic	13 (65.00)	16 (69.57)	
Hemiplegic side			0.711
Left	11 (55.00)	15 (65.22)	
Right	9 (45.00)	8 (34.78)	
Functional scores			
FAC	1.00 [0.00–1.00]	1.00 [0.00–1.00]	0.646
FAC			0.809
0	7 (35.00)	9 (39.13)	
1	10 (50.00)	12 (52.17)	
2	3 (15.00)	2 (8.70)	
TCT	60.30 ± 30.07	50.96 ± 33.50	0.345
K-MMSE	21.05 ± 8.18	20.00 ± 7.31	0.659
K-MBI	29.50 ± 21.58	27.09 ± 21.09	0.713
BBS	9.85 ± 13.12	8.39 ± 10.84	0.692
FMA	16.10 ± 9.86	13.96 ± 11.75	0.519

Values are presented as *n* (%), mean ± standard deviation, or median [interquartile range]

*BMI* body mass index, *FAC* Functional Ambulatory Category, *TCT* Trunk Control Test, *K-MMSE* Korean Mini Mental State Examination, *K-MBI* Korean Modified Barthel Index, *BBS* Berg Balance Scale, *FMA* Fugl-Meyer Assessment

### Functional ambulation category score

#### Baseline and postintervention comparisons

Observed FAC scores at baseline and postintervention are summarized in Table 2. FAC scores increased in both groups after the intervention period, with a higher postintervention FAC score in the R-BoT + plus group than in the control group (*p* = 0.043; Table 2).

**Table 2:** Differences in outcome measure scores between the R-BoT + plus and control groups

Outcome measure	Pre-intervention	Post-intervention
FAC		
R-BoT + plus	0.80 ± 0.70	2.35 ± 1.76
Control	0.70 ± 0.63	1.26 ± 1.18
*p* value	0.646	0.043
TCT		
R-BoT + plus	60.30 ± 30.07	92.25 ± 15.82
Control	50.96 ± 33.50	73.26 ± 33.06
*p* value	0.341	0.020
K-MMSE		
R-BoT + plus	21.05 ± 8.18	26.30 ± 8.61
Control	20.00 ± 7.31	22.78 ± 4.74
*p* value	0.662	0.115
K-MBI		
R-BoT + plus	29.50 ± 21.58	54.20 ± 25.72
Control	27.09 ± 21.09	47.43 ± 23.93
*p* value	0.714	0.38
BBS		
R-BoT + plus	9.85 ± 13.12	28.47 ± 17.94
Control	8.39 ± 10.84	23.52 ± 17.95
*p* value	0.696	0.379
FMA		
R-BoT + plus	17.40 ± 8.64	20.62 ± 9.95
Control	14.12 ± 12.92	19.57 ± 11.60
*p* value	0.412	0.803

Values are presented as mean ± standard deviation

*Pre* preintervention, *Post* postintervention, *FAC* Functional Ambulatory Category, *TCT* Trunk Control Test, *K-MMSE* Korean Mini Mental State Examination, *K-MBI* Korean Modified Barthel Index, *BBS* Berg Balance Scale, *FMA* Fugl-Meyer Assessment

#### Change scores from baseline to postintervention

In the pre–post change-score analysis, FAC improvement was greater in the R-BoT + plus group than in the control group (*p* = 0.010; Table 3), supporting greater improvement in gait independence in the R-BoT + plus group.

**Table 3:** Preintervention-to-postintervention change in outcome measure score between the two groups at two different time points

Outcome measure	R-BoT + plus	Control	*p* value	FDR *q*-value
FAC (primary)	1.55 ± 1.32	0.57 ± 0.79	0.010	–
TCT	31.95 ± 22.37	22.30 ± 25.78	0.102	0.225
K-MMSE	5.25 ± 7.31	2.78 ± 4.86	0.140	0.225
K-MBI	24.70 ± 14.70	20.35 ± 15.86	0.100	0.225
BBS	20.47 ± 14.53	15.13 ± 14.61	0.180	0.225
FMA	5.95 ± 6.34	6.35 ± 6.15	0.909	0.909

Values are presented as mean ± standard deviation. Change scores were calculated as postintervention minus baseline values. Between-group comparisons were performed using the Wilcoxon rank-sum test. FDR *q*-values were calculated for secondary outcomes using the Benjamini–Hochberg procedure to account for multiple comparisons. FAC change-score analysis was considered supportive of the primary analysis based on the linear mixed-effects model

*BBS* Berg Balance Scale, *FAC* Functional Ambulation Category, *FDR* false discovery rate, *FMA* Fugl-Meyer Assessment, *K-MBI* Korean version of the Modified Barthel Index, *K-MMSE* Korean Mini-Mental State Examination, *SD* standard deviation, *TCT* Trunk Control Test

#### Longitudinal FAC outcomes through 3-month follow-up: mixed-effects model and Sankey diagrams

**Fig. 3:**
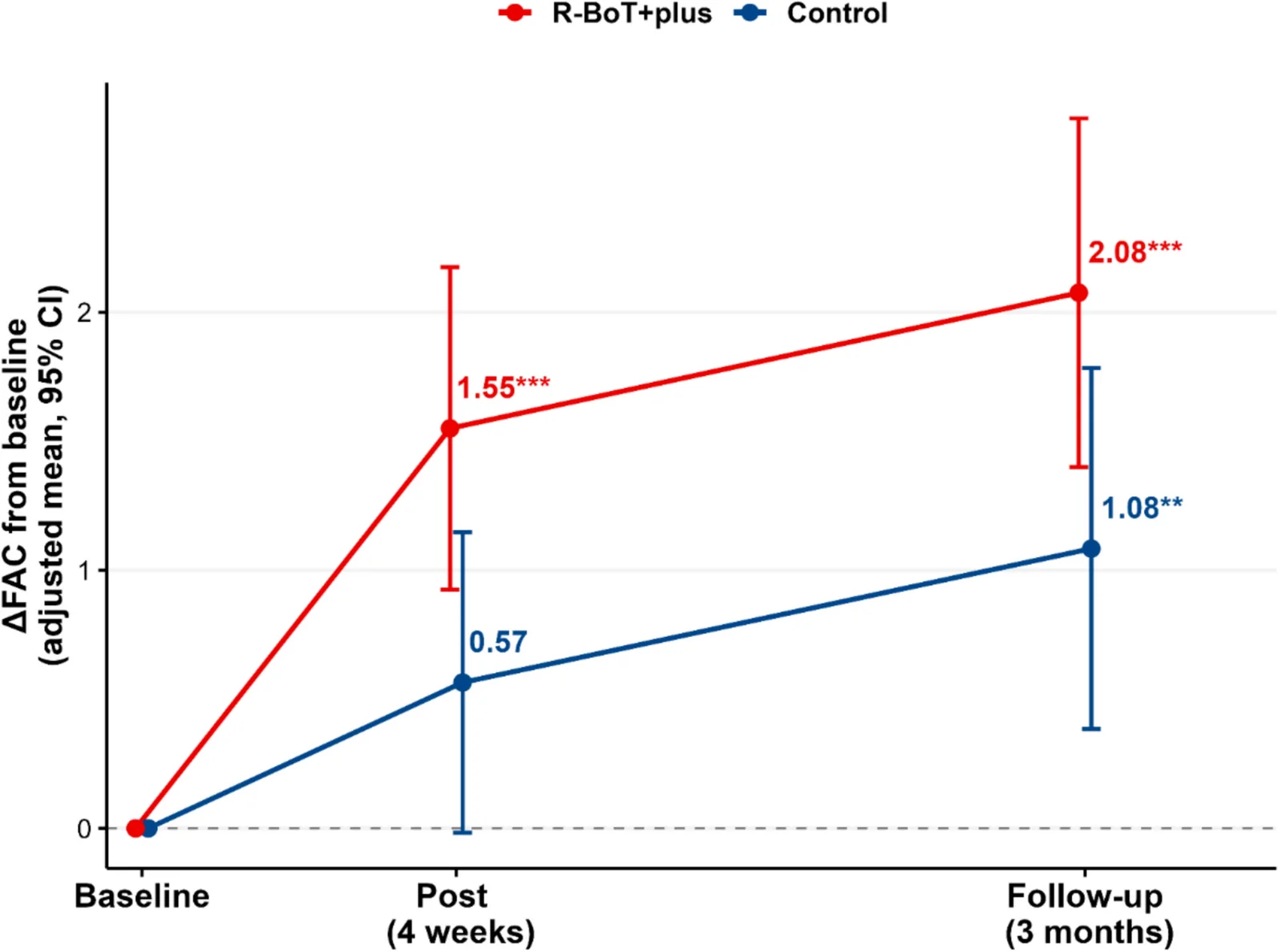
Model-estimated changes from baseline in FAC scores at postintervention and 3-month follow-up. * Statistically significant within-group change from baseline at each time point. ΔFAC, change in Functional Ambulation Category score; CI, confidence interval; Post, postintervention

In the primary linear mixed-effects model incorporating FAC at baseline, postintervention, and 3-month follow-up, the overall group-by-time interaction was statistically significant (*χ*^2^ = 8.54, *df* = 2, *p* = 0.014; Table 4), indicating that the trajectory of FAC improvement differed between groups. Model-estimated FAC scores increased from 0.80 ± 0.30 at baseline to 2.35 ± 0.30 at postintervention and 2.88 ± 0.33 at 3-month follow-up in the R-BoT + plus group, and from 0.70 ± 0.28 to 1.26 ± 0.28 and 1.78 ± 0.33, respectively, in the control group. In post hoc model-based contrasts, the R-BoT + plus group showed greater improvement from baseline than the control group at postintervention (difference in change, 0.98; 95% CI 0.23–1.74; Holm-adjusted *p* = 0.022) and at 3-month follow-up (difference in change, 0.99; 95% CI 0.14–1.85; Holm-adjusted *p* = 0.024) (Table 4; Fig. 3). These findings indicate a consistent pattern of greater FAC improvement over time in the experimental group than in the control group.

**Table 4:** Model-estimated FAC scores and changes from baseline based on the linear mixed-effects model

Time point	Model-estimated FAC score^a^		Change from baseline^b^		Difference in change^c^	
	R-BoT + plus	Control	R-BoT + plus	Control	R-BoT + plus—Control, 95% CI	Holm-adjusted *p* value^d^
Baseline (pre-intervention)	0.80 ± 0.30	0.70 ± 0.28	–	–	–	–
Postintervention (4 weeks)	2.35 ± 0.30	1.26 ± 0.28	1.55 ± 0.28	0.57 ± 0.26	0.98 (0.23, 1.74)	0.022
Follow-up (3 months)	2.88 ± 0.33	1.78 ± 0.33	2.08 ± 0.30	1.08 ± 0.31	0.99 (0.14, 1.85)	0.024

*FAC* Functional Ambulation Category, *CI* confidence interval

Overall time × group interaction was statistically significant (*χ*^2^ = 8.54, *df* = 2, *p* = 0.014)

^a^Values are model-estimated means ± standard errors derived from the linear mixed-effects model

^b^Change from baseline was estimated from the same model

^c^Difference in change was calculated as the change from baseline in the R-BoT + plus group minus the change from baseline in the control group; positive values indicate greater improvement in the R-BoT + plus group

^d^*p* values were adjusted for multiple comparisons using the Holm method for post hoc between-group contrasts of change from baseline at each follow-up time point

**Fig. 4:**
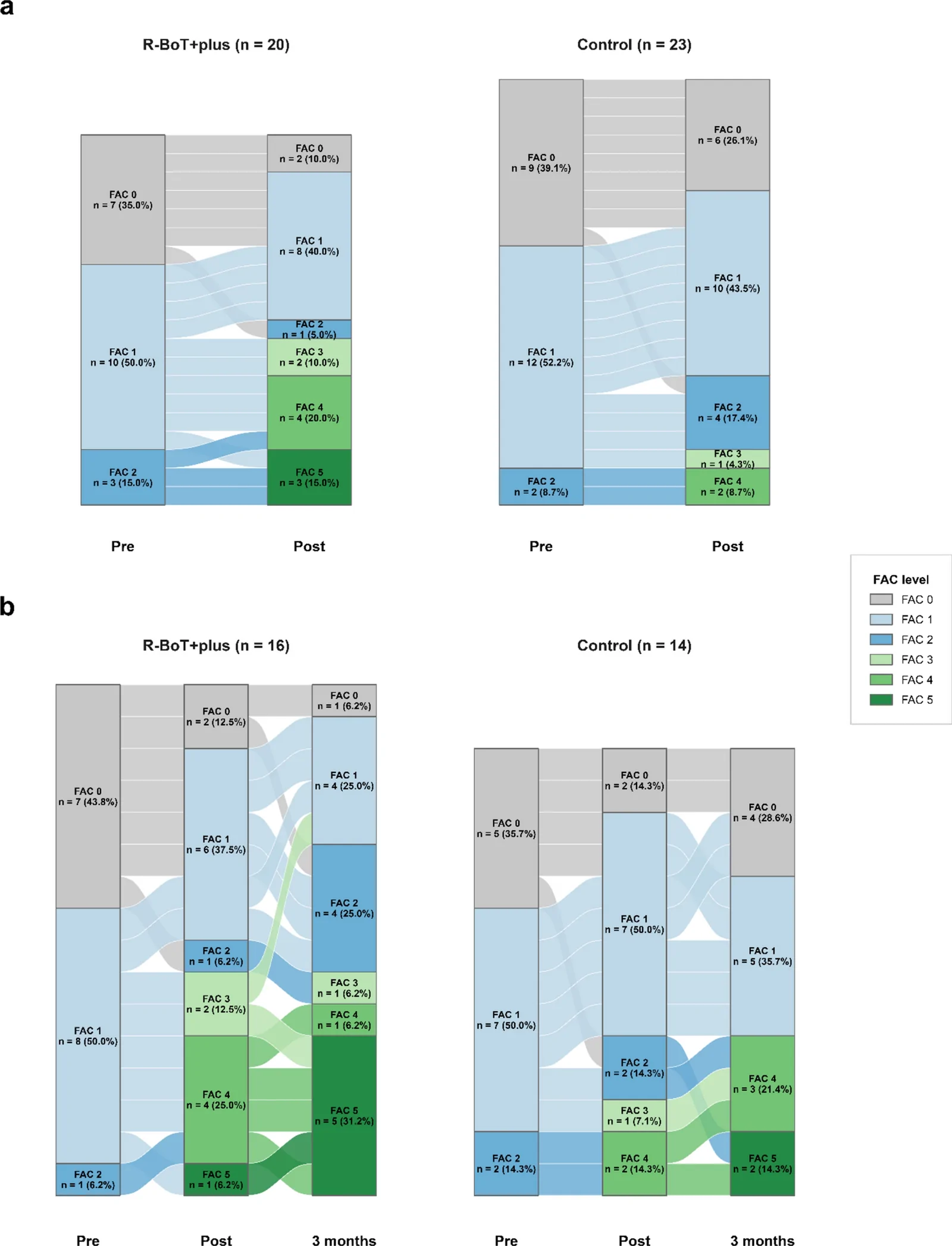
Sankey diagrams illustrating individual trajectories of the FAC scores **a** from baseline to postintervention and **b** from baseline to postintervention and 3-month follow-up in the R-BoT + plus and control groups. The pre–post Sankey diagram was based on participants with available FAC data at both baseline and postintervention (R-BoT + plus, *n* = 20; control, *n* = 23), whereas the three-time-point Sankey diagram was based on participants with available FAC data at baseline, postintervention, and 3-month follow-up (R-BoT + plus, *n* = 16; control, *n* = 14). FAC, Functional Ambulation Category; Pre, preintervention (baseline); Post, postintervention

Figure 4a presents the Sankey diagrams illustrating individual-level trajectories of FAC from baseline (preintervention) to post-intervention in both groups, providing a visual representation of the direction and magnitude of change in gait independence for each participant. When comparing preintervention and postintervention FAC levels, a greater proportion of participants in the R-BoT + plus group demonstrated improvements of two or more FAC levels compared with the control group. Notably, recovery to the highest level of gait independence (FAC 5) was observed exclusively in the R-BoT + plus group.

Because the number of participants with available 3-month follow-up data was limited, a separate Sankey diagram was constructed to depict FAC transitions from baseline to postintervention and to 3-month follow-up (Fig. 4b). Despite the smaller sample size, this longitudinal visualization showed a similar pattern: participants in the R-BoT + plus group exhibited a greater tendency toward sustained, progressive improvements in FAC than those in the control group.

In the FAC change-category analysis corresponding to the Sankey diagrams, a greater proportion of participants in the R-BoT + plus group achieved ≥2-level improvement from baseline to postintervention compared with the control group (50.0% vs. 17.4%), showing a directionally favorable but statistically non-significant pattern (*p* = 0.063). The between-group distribution of FAC change categories from baseline to 3-month follow-up was also not statistically significant (*p* = 0.230) (Supplementary Table S1).

### TCT score

Changes in TCT scores from baseline to postintervention are summarized in Table 3. Although the mean improvement in TCT was numerically greater in the R-BoT + plus group than in the control group (31.95 ± 22.37 vs. 22.30 ± 25.78), the between-group difference in change scores did not reach statistical significance after FDR adjustment (*p* = 0.102; FDR *q* = 0.225). To further elucidate potential heterogeneity in treatment effects by baseline trunk control severity, exploratory robust regression analysis was conducted.

#### Interaction between treatment group and baseline severity

The model used postintervention TCT score as the dependent variable and included treatment group, baseline TCT score, and their interaction as independent variables.

The robust regression model revealed a significant interaction between the treatment group and baseline TCT scores (*β* = −0.31; 95% CI −0.60 to −0.03; *t* = −2.172; *p* = 0.036), indicating that baseline TCT scores significantly modified the treatment effect. The negative interaction coefficient suggests that the superiority of the R-boT + plus group over the control group progressively decreased as baseline TCT scores increased (i.e., as initial severity decreased). At the theoretical baseline TCT of 0, the estimated group main effect was 31.36 points (95% CI 13.11–49.62; *p* = 0.002; Table 5A).

**Table 5:** Regression analysis of post-intervention TCT and comparison of treatment effects across baseline TCT levels

(A) Robust regression analysis for postintervention TCT: interaction between treatment group and baseline severity			
Variable	Coefficient (95% CI)	*t*-value	*p* value
Group (treatment vs. control)	31.36 (13.11, 49.62)	8.308	0.002
Baseline TCT (per 1 point)	0.58 (0.39, 0.76)	6.226	<0.001
Group × baseline TCT	−0.31 (−0.60, −0.03)	−2.172	0.036
The dependent variable was postintervention TCT score. Independent variables were treatment group, baseline TCT score, and the group × baseline TCT interaction. *β* for group is the difference at baseline TCT = 0. The group × baseline TCT term represents effect modification by baseline severity			

(B) Comparison of treatment effects (treatment–control) at different baseline TCT levels			
Baseline TCT (preintervention)	Treatment–control (estimate, 95% CI)	*t*-value	*p* value
25	23.52 (10.58, 36.45)	3.678	<0.001
50	15.67 (6.38, 24.96)	3.413	0.002
75	7.83 (−2.76, 18.41)	1.647	0.108
Treatment effects are presented as R-BoT + plus minus control. The baseline TCT values were selected to represent clinically relevant baseline severity levels			

*TCT* Trunk Control Test, *CI* confidence interval

#### Conditional treatment effects by baseline level

The intervention’s efficacy was meaningfully moderated by baseline functional status, with a more pronounced therapeutic response among participants with greater initial impairment. Within the severe-to-moderate range of trunk control (TCT score, ~ 0–50) [21], the treatment group demonstrated the largest and statistically significant gains. The estimated experimental minus control difference was greatest at a baseline TCT of 25 (23.52 points; 95% CI 10.58–36.45; *p* < 0.001) and remained robust at the moderate impairment threshold of TCT 50 (15.67 points; 95% CI 6.38–24.96; *p* = 0.002). However, as baseline scores approached the 75-point threshold—a clinically recognized predictor of achieving independent ambulation [21]—the between-group difference progressively diminished and no longer reached statistical significance, declining to 7.83 points at a baseline TCT of 75 (95% CI −2.76 to 18.41; *p* = 0.108; Table 5B).

**Fig. 5:**
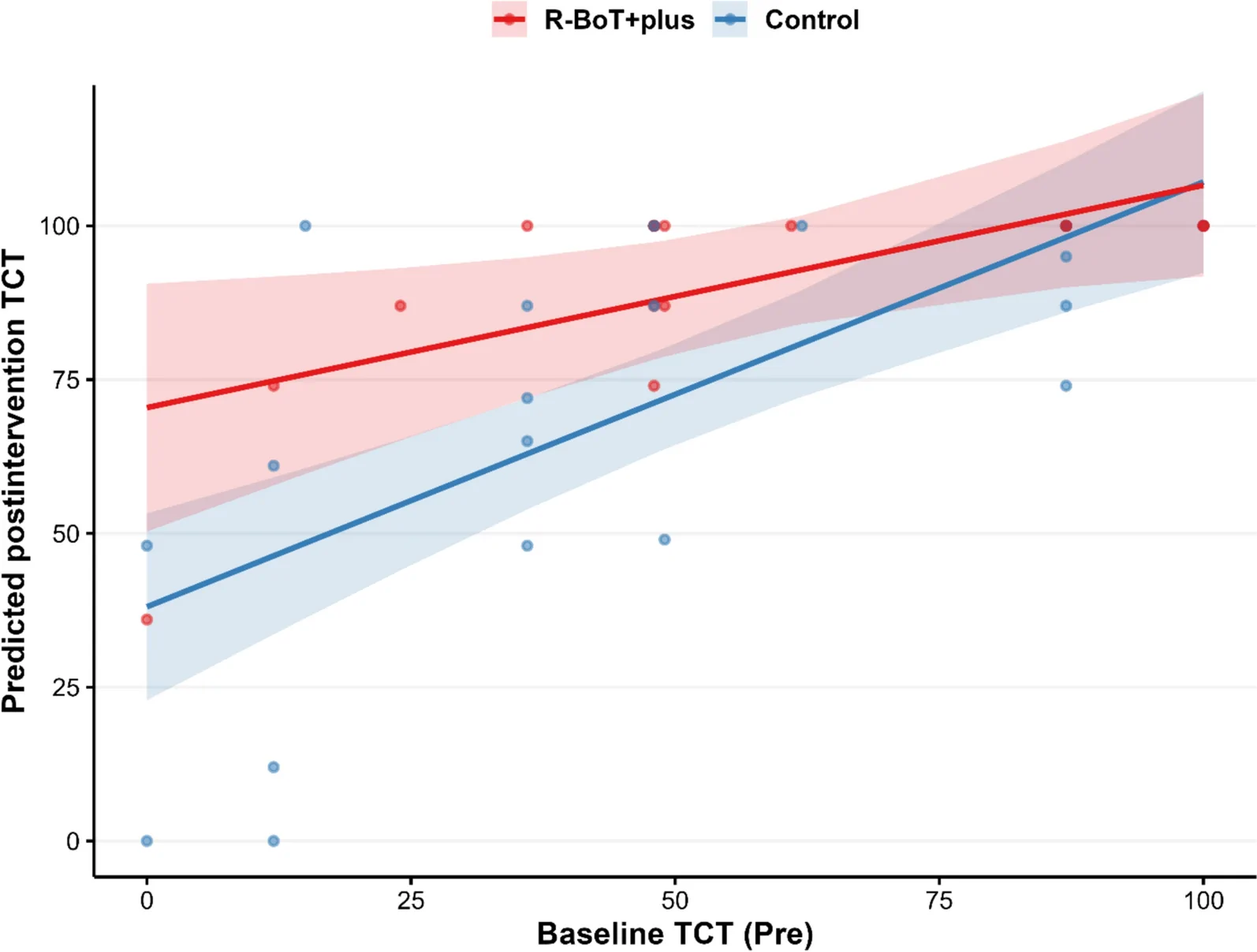
Predicted postintervention TCT score by baseline TCT score in the R-BoT + plus and control groups. TCT, Trunk Control Test

Taken together, these findings indicate a potential ceiling effect among patients with higher baseline trunk control and suggest that R-BoT + plus provides its greatest clinical benefit in individuals who have not yet reached the functional threshold for independent walking, as illustrated in Fig. 5, which shows a larger treatment effect at lower baseline TCT levels that progressively diminishes as baseline trunk control increases.

### Other secondary outcomes

K-MMSE, K-MBI, BBS, and lower-extremity FMA scores improved from baseline to postintervention in both groups (Table 3). Although the experimental group generally demonstrated numerically greater improvements in cognitive function, activities of daily living, and balance outcomes, between-group differences in change scores were not statistically significant. Similarly, lower-extremity FMA scores improved substantially in both groups without a significant between-group difference.

### Adverse events

Adverse events were monitored throughout the intervention period. In the R-BoT + plus group, one participant sustained a mild bruise on the thigh, which was considered clearly related to the intervention; however, the participant continued the study without interruption. Another participant developed mild spasticity of the left upper extremity, which was judged to be unrelated to the intervention and did not require discontinuation.

The lesion size during the study period increased in one participant; this event was considered unrelated to the intervention and the patient was discontinuation from the study. In addition, one participant developed a pulmonary thromboembolism (PTE), which was classified as a serious adverse event. The causal relationship to the intervention was deemed unassessable, and the intervention was temporarily suspended. No serious adverse events were judged to be definitively related to the R-BoT + plus device.

## Discussion

This randomized controlled trial in patients with subacute stroke demonstrated that adding R-BoT + plus verticalization-based robotic locomotor training to conventional rehabilitation resulted in significantly greater improvements in gait independence than conventional rehabilitation alone [22, 23]. In the primary longitudinal analysis, the group-by-time interaction for FAC was statistically significant, indicating that the trajectory of gait independence differed between groups over time. Post hoc model-based contrasts further showed greater improvement from baseline in the R-BoT + plus group at both postintervention and 3-month follow-up after adjustment for multiple comparisons. The supportive pre–post change-score analysis also favored the R-BoT + plus group, consistent with the longitudinal mixed-effects model findings.

Although unadjusted change scores of the TCT did not show a significant between-group after multiplicity adjustment, exploratory baseline-adjusted robust regression suggested that the treatment effect of R-BoT + plus on postintervention trunk control may vary according to baseline trunk control severity. This finding underscores the substantial heterogeneity in baseline trunk control among patients in the subacute phase after stroke and highlights the importance of accounting for initial functional status when evaluating intervention effects. Recovery of trunk control after stroke may follow nonlinear trajectories, with greater improvements often observed in individuals with more severe initial impairment, whereas patients with relatively preserved trunk function may demonstrate reduced responsiveness because of ceiling effects [22, 24]. By incorporating baseline TCT and the group-by-baseline interaction into a robust regression model, the present analysis demonstrated that the beneficial effect of R-BoT + plus on trunk control was most pronounced among participants with lower baseline TCT scores, indicating a robust intervention effect on proximal postural control in patients with greater initial impairment. In contrast, between-group differences diminished as baseline trunk control increased, suggesting limited additional benefit beyond conventional rehabilitation in patients with higher baseline function. These findings indicate that simple change score analyses may obscure clinically relevant, baseline-dependent treatment effects and support the use of baseline-adjusted analytic approaches when assessing trunk-related outcomes in heterogeneous stroke populations.

The neurophysiological characteristics of the R-BoT + plus intervention may explain the preferential effects on trunk control and ambulation. Early verticalization with graded weight-bearing provides intensive afferent input from the lower limbs and trunk, facilitating activation of spinal and supraspinal locomotor networks [25, 26]. Repetitive, bilateral stepping-like movements may engage central pattern generator-related circuits and promote interlimb coordination while simultaneously challenging axial stability [26, 27]. Although the R-BoT + plus system does not involve forward progression typical of conventional RAGT systems, it can be conceptualized as a form of robot-assisted verticalization-based early locomotor training for patients with severe gait impairment. The system facilitates locomotor-like cyclic lower-limb movements that provide task-related sensory input and repetitive afferent stimulation. Such training may serve as an important neurophysiological preparation for subsequent gait recovery by activating locomotor-related neural circuits in patients who are unable to tolerate conventional upright gait training during the early stages of rehabilitation. In addition, FES can augment muscle activation and sensory feedback, reinforcing sensorimotor coupling and activity-dependent plasticity [28]. Real-time visual feedback delivered through task-oriented virtual environments may further enhance motor learning by increasing patient engagement, attention, and error-based correction during training [29, 30].

FAC is an ordinal, milestone-based scale reflecting discrete levels of walking independence rather than continuous quantitative change [25, 31]. Previous studies have demonstrated that transitions between FAC categories represent clinically meaningful changes in gait independence and functional ambulation after stroke [32]. Accordingly, statistically significant between-group differences in FAC may reflect clinically meaningful transitions in walking ability, even when numerical changes appear modest. In this context, the observed significant improvement in FAC in the R-BoT + plus group is consistent with both the longitudinal trajectory analysis and the sustained between-group differences observed over time, supporting the clinical relevance of the intervention.

Although exploratory baseline-adjusted analyses suggested a greater treatment effect on trunk control in patients with lower baseline TCT scores, no significant between-group difference was observed for balance measured by the BBS. This discrepancy may reflect differences in measurement constructs, as many BBS items require independent standing and dynamic balance, limiting responsiveness in patients with severe gait impairment and low baseline FAC levels. As a result, BBS scores in this study may have been subject to a floor effect, reducing sensitivity to detect treatment-related changes. In contrast, the TCT emphasizes bed mobility and sitting balance, making it more suitable for assessing early postural control in patients with severe functional limitations.

Future studies should examine the effects of R-BoT + plus training in patient populations with higher baseline ambulatory function, where balance measures such as the BBS may be more sensitive to change. Stratified analyses by baseline FAC or the use of balance assessments tailored to early rehabilitation stages may further clarify the relationships among trunk control, balance, and gait recovery.

Cognitive function, ADL, and broad lower-extremity motor function outcomes improved substantially in both groups, reflecting the combined effects of spontaneous neurological recovery and comprehensive inpatient rehabilitation during the subacute phase.

Some limitations warrant consideration. First, the total therapy dose differed between groups, as participants in the experimental group received additional R-BoT + plus training alongside conventional rehabilitation. Accordingly, the observed effects may partly reflect increased therapy intensity rather than the isolated effect of the robotic intervention. Second, because of the nature of the device-based rehabilitation intervention, participants and treating therapists could not be blinded to group allocation. This may have introduced performance bias or expectancy effects that could have influenced treatment engagement or rehabilitation outcomes. However, allocation concealment was maintained, and outcome assessments were conducted by assessors who were not involved in treatment delivery and were instructed to remain unaware of group allocation whenever feasible, in an effort to minimize assessment bias. Third, although the sample size was calculated based on the planned primary FAC analysis, the overall sample size remained relatively small, limiting statistical power for secondary outcome analyses despite the use of FDR *q*-values to address multiplicity. Fourth, missing outcome data were not imputed, and a strict intention-to-treat analysis was not performed; thus, potential bias related to dropout or missing follow-up data cannot be completely excluded. Fifth, quantitative robotic training-dose parameters such as total step count or cadence were not systematically recorded. Because the study population consisted of patients with severe gait impairment who required individualized combinations of trunk control training, supported standing, gradual verticalization, and therapist-assisted stepping activities, it was difficult to define or standardize a uniform gait-training dose across participants; thus, the relationship between training intensity and clinical outcomes could not be fully evaluated. Finally, the single-center design may limit the generalizability of the findings. In addition, the implementation of robotic-assisted verticalization training requires specialized equipment, therapist supervision, and additional intervention time, which may increase rehabilitation-related costs compared with conventional rehabilitation alone. Therefore, future studies examining cost-effectiveness, resource utilization, and long-term functional outcomes will be important to determine the broader clinical applicability and practical value of R-BoT + plus in real-world rehabilitation settings.

Importantly, the present findings should be interpreted as evidence for the incremental benefit of adding R-BoT + plus training to an existing rehabilitation program rather than as evidence for superiority of the robotic intervention alone. Because the experimental group received additional therapy beyond conventional rehabilitation, the observed effects likely reflect the combined influence of robotic verticalization-based locomotor training and increased rehabilitation exposure. Despite these limitations, the present findings support the feasibility and potential clinical value of incorporating R-BoT + plus verticalization-based robotic locomotor training into early rehabilitation programs for patients with subacute stroke and severe gait impairment. By targeting key neurophysiological prerequisites for walking, including trunk stability, sensorimotor integration, and task-specific afferent input during upright activity, R-BoT + plus may complement conventional rehabilitation and facilitate earlier recovery of gait independence.

## Conclusions

In this randomized controlled trial, adding R-BoT + plus verticalization-based robotic locomotor training to conventional rehabilitation resulted in a statistically significant improvement in gait independence compared with conventional rehabilitation alone in patients with subacute stroke. In addition, baseline-adjusted analyses demonstrated that R-BoT + plus conferred greater benefits in trunk control, particularly among patients with lower baseline trunk control, indicating a targeted effect on proximal postural stability in individuals with greater initial impairment. Although improvements in cognitive function, balance, ADL, and lower-extremity motor function were observed in both groups, the preferential effects on gait independence and trunk control suggest that R-BoT + plus provides complementary benefits by addressing key neurophysiological components of gait recovery.

## Supplementary Information

Additional file 1.

## Data Availability

The data presented in this study are available on request from the corresponding author. The data are not publicly available due to privacy matters.

## Abbreviations

ADL: Activities of daily living
BBS: Berg Balance Scale
FAC: Functional Ambulation Category
FES: Functional electrical stimulation
FMA: Fugl-Meyer Assessment
K-MBI: Korean Modified Barthel Index
K-MMSE: Korean Mini-Mental State Examination
MMT: Manual Muscle Test
RAGT: Robot-assisted gait training
TCT: Trunk Control Test
