# Supplementary material for: Effects of adding R-BoT + plus verticalization-based robotic locomotor training to conventional rehabilitation in patients with subacute stroke: a randomized controlled trial
Source: J Neuroeng Rehabil. 2026 Jun 23;23:265. doi: 10.1186/s12984-026-02053-z (PMC13563739; doi:10.1186/s12984-026-02053-z)
Supplement: Supplementary file 1 [file 12984_2026_2053_MOESM1_ESM.docx]

**Supplementary Table S1.** FAC change category analysis corresponding to the Sankey diagrams

| **Time comparison** | **FAC change category** | **R-BoT+plus** | **Control** | **p-Value** |
| --- | --- | --- | --- | --- |
| Baseline to postintervention | ≤ −1 | 0 (0.00) | 0 (0.00) | 0.063 |
|  | 0 | 6 (30.00) | 14 (60.87) |  |
|  | +1 | 4 (20.00) | 5 (21.74) |  |
|  | ≥ +2 | 10 (50.00) | 4 (17.39) |  |
| Baseline to 3-month follow-up | ≤ −1 | 0 (0.00) | 2 (14.29) | 0.230 |
|  | 0 | 3 (18.75) | 5 (35.71) |  |
|  | +1 | 3 (18.75) | 2 (14.29) |  |
|  | ≥ +2 | 10 (62.50) | 5 (35.71) |  |

Values are presented as n (%). FAC change was categorized as ≥+2, +1, 0, and ≤−1. Between-group distributions of FAC change categories were compared using Fisher’s exact test because of sparse cell counts.

FAC, Functional Ambulation Category.
